# Automated detection of methicillin-resistant *Staphylococcus aureus* with the MRSA CHROM imaging application on BD Kiestra Total Lab Automation System

**DOI:** 10.1128/jcm.01445-23

**Published:** 2024-04-01

**Authors:** Erin McElvania, Susan Mindel, Jaap Lemstra, Karin Brands, Parul Patel, Caryn E. Good, Didier Morel, Cedrick Orny, Jean-Marc Volle, Marc Desjardins, Daniel Rhoads

**Affiliations:** 1 Northshore University Health System, Evanston, Illinois, USA; 2 Becton, Dickinson and Company– Integrated Diagnostic Solutions, Sparks, Maryland, USA; 3 BD Kiestra BV, Drachten, the Netherlands; 4 University Hospitals Cleveland Medical Center, Cleveland, Ohio, USA; 5 Becton, Dickinson and Company – HEOR & RWE Data Science, Eybens Isere, France; 6 Becton, Dickinson and Company – Innovation Software Engineering, Eybens Isere, France; 7 Eastern Ontario Regional Laboratory Association, Ottawa, Ontario, Canada; 8 Cleveland Clinic, Cleveland, Ohio, USA; Mayo Clinic, Jacksonville, Florida, USA

**Keywords:** methicillin-resistant *Staphylococcus aureus *(MRSA), lab automation, BD Kiestra

## Abstract

The virulence of methicillin-resistant *Staphylococcus aureus* (MRSA) and its potentially fatal outcome necessitate rapid and accurate detection of patients colonized with MRSA in healthcare settings. Using the BD Kiestra Total Lab Automation (TLA) System in conjunction with the MRSA Application (MRSA App), an imaging application that uses artificial intelligence to interpret colorimetric information (mauve-colored colonies) indicative of MRSA pathogen presence on CHROMagar chromogenic media, anterior nares specimens from three sites were evaluated for the presence of mauve-colored colonies. Results obtained with the MRSA App were compared to manual reading of agar plate images by proficient laboratory technologists. Of 1,593 specimens evaluated, 1,545 (96.98%) were concordant between MRSA App and laboratory technologist reading for the detection of MRSA growth [sensitivity 98.15% (95% CI, 96.03, 99.32) and specificity 96.69% (95% CI, 95.55, 97.60)]. This multi-site study is the first evaluation of the MRSA App in conjunction with the BD Kiestra TLA System. Using the MRSA App, our results showed 98.15% sensitivity and 96.69% specificity for the detection of MRSA from anterior nares specimens. The MRSA App, used in conjunction with laboratory automation, provides an opportunity to improve laboratory efficiency by reducing laboratory technologists’ labor associated with the review and interpretation of cultures.

## INTRODUCTION

As one of the most common causative organisms of healthcare-associated infections (1), methicillin-resistant *Staphylococcus aureus* (MRSA) is of particular concern in hospital settings. Nasal colonization by MRSA is a widely recognized risk factor for the development of hospital-acquired MRSA infections and transmission to other individuals (2, 3), and about 5% of inpatient populations carry MRSA in their normal nasal flora (4). Moreover, while many asymptomatic individuals have nasal colonization with MRSA (5), an array of complicating medical factors significantly increase patients’ risk of developing an invasive infection (5–7), including use of central lines, presence of indwelling hardware, skin and wound manipulations, and intubation. Hospital-acquired MRSA can present with symptoms that vary by body site and severity, but severe infections including bacterial sepsis, surgical site infections, and ventilator-associated pneumonia are associated with high morbidity and mortality. In 2017, approximately 323,700 MRSA infections were reported in US inpatient facilities accounting for 10,600 deaths (8).

Traditionally, MRSA colonization was detected by performing bacterial culture followed by susceptibility testing of any *S. aureus* identified to determine whether the isolate was methicillin resistant or by PCR detection of the staphylococcal chromosomal cassette (SCCmec cassette) containing the microbial electrolysis cell *A* (*mecA*) gene along with other *S. aureus*-specific gene targets from nasal swab specimens. More recently, chromogenic agar has been developed which exploits differences in pathogen metabolism and uses chromogens that result in pigmented colonies when the target organism is present in culture. The presence of pigmented colonies allows technologists to easily identify the target organism on agar plates. The use of chromogenic agar allows laboratories to detect targeted organisms such as MRSA more rapidly and saves labor by eliminating the requirement for biochemical or matrix-assisted laser desorption/ionization-time of flight (MALDI-TOF) identification and susceptibility testing. Pigmented colonies are also ideal for detection by artificial intelligence (AI) programs using digital images of agar plates.

**Fig 1:**
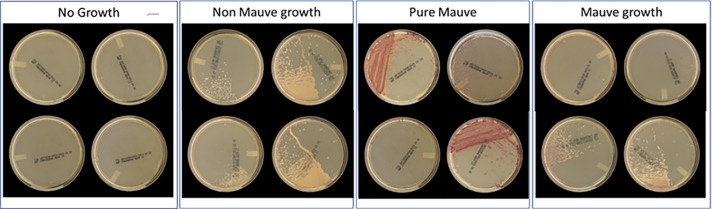
Examples of agar plates by batch sorting categories (no growth, non-mauve growth, pure mauve, and mixtures of mauve and non-mauve growth).

The BD Kiestra Total Lab Automation (TLA) System (Becton, Dickinson and Company; Sparks, MD, USA) automates repetitive, manual tasks traditionally performed by clinical laboratory technologists such as media selection, labeling, specimen inoculation, agar plate streaking, and transporting agar plates to and from incubators. Kiestra TLA is programmed to automatically image agar plates at predefined periods of incubation. These images are then digitally viewed and interpreted by laboratory technologists after which results are entered into the laboratory information system to be viewed by physicians and clinical staff in the electronic medical record. The BD Kiestra Methicillin-resistant *Staphylococcus aureus* Application (herein defined as MRSA App) (Becton, Dickinson and Company, Sparks, MD, USA) is an artificial intelligence program that works in conjunction with the Kiestra TLA system to detect MRSA colony growth from digital images of BD BBL CHROMagar MRSA II (CHROMagar) (Becton, Dickinson and Company; Sparks, MD, USA). Using Kiestra TLA, specimens from the anterior nares are inoculated to CHROMagar, incubated, and imaged using the MRSA App. The MRSA App uses digital images to detect the presence of colony growth with the appropriate colorimetric interpretation required for the identification of MRSA and then sorts cultures into designated categories for batch reading (Fig. 1). This is the first investigation evaluating the accuracy of the MRSA App in identifying MRSA *via* colorimetric detection (mauve-colored colonies) compared to standard of care (i.e., manual reading of agar plate images by proficient laboratory technologists).

## MATERIALS AND METHODS

In our investigation, the BD Kiestra TLA system was used in conjunction with the MRSA App, an imaging application that uses artificial intelligence to interpret colorimetric information (mauve-colored colonies) indicative of MRSA pathogen presence on CHROMagar chromogenic media. As part of this study, a Colony Information System (*CIS*) was also used to provide a comprehensive platform that managed and analyzed colony information throughout the microbiology process.

### Specimen collection

Enrollment of remnant MRSA anterior nares surveillance Eswab specimens took place from January to August 2019 at three sites, two in the United States and one in Canada. All Eswab specimens were collected according to the standard of care used at each facility and only one Eswab per patient was eligible for inclusion in the study. Specimens were excluded if plates were removed from the incubator at any point prior to the completion of the imaging process or if specimens met rejection criteria based on routine laboratory protocols. Deidentified participant information, including patient age and gender, was stored in Clindex (BD’s Clinical Information system) and the *CIS*. All sites received approval for the study protocol from their respective Institutional Review Board (IRB) prior to study initiation. This manuscript was prepared using the Standards for Reporting Diagnostic Accuracy (STARD) guidelines.

### MRSA App artificial intelligence program

The MRSA App employs conventional image processing techniques to discern areas of interest within the Petri dish images obtained post-incubation. These areas represent regions of the images where pixel values have undergone alterations during the incubation period. Typically, these zones include colony forming units (CFUs) or incidental artifacts such as dust, agar cracks, etc. Distinct features, including color, morphology, metrics, and opacity, are extracted from each zone and are subsequently fed into an initial machine-learning model that classifies each zone as either a CFU or another entity. Plate-level metrics, along with descriptors characterizing the population of detected CFUs, are used as input features for a second model that assesses the growth status of the plate. Next, the first model’s identification of each CFU is classified by a third model that determines whether it is a mauve or a non-mauve CFU. Descriptors characterizing the population of mauve CFUs are then used as input features for a fourth model which reports plate positivity based on the presence of the mauve-colored colonies.

### Specimen processing

Enrolled study samples were deidentified and loaded in the *CIS* within 48 hours of collection using BD Kiestra analysis parameters. 30 μl of the Eswab specimen was inoculated onto CHROMagar using InoqulA (with streak patterns 19 and 20) and incubated for 22 hours ± 30 min, at 35°C ± 2°C. Images captured between hour 20 and hour 24 of incubation (herein referred to as 24 hours) were evaluated by the MRSA App for the presence of colony growth and colorimetric colonies (mauve) associated with the presence of MRSA pathogens. The 2-hour allowance before and after the 22-hour incubation period for image acquisition, which is aligned with the information provided in the Users’ Manual, was intended to accommodate variations in system workload at each site which could slightly impact the exact timing of the image acquisition process. Results were subsequently compared to the reference standard for evaluation which consists of manual reading of the same digital images by trained laboratory technologists and agreement rates between the two techniques were evaluated.

### Plate reading

All plate images acquired at 24-hour incubation were read by two laboratory technologists at each site who were blinded to each other’s results as well as those generated by the MRSA App. If consensus on the result was not reached, a third trained reader was used as an arbiter. Plate images were assigned to growth/no growth categories and those showing colony growth were further assessed for identification of chromogenic colony appearance signaling the presence of MRSA microorganisms. MRSA App and technologist read results obtained with each method were subsequently compared, using the technologist read results as the reference standard. All technologists involved in the reading of digital plate images received training prior to study initiation to ensure accuracy in the identification of colony growth and MRSA-pigmented colony growth. On each day of testing, quality control was performed using *S. aureus* ATCC 43300 or ATCC 33591 as the MRSA-positive control and *Enterococcus faecalis* ATCC 29212 as a negative control.

### Statistical analysis

The performance of the MRSA App was assessed following FDA *Statistical Guidance on Reporting Results from Studies Evaluating Diagnostic Tests* (UCM 071287, 2007) (9). Sensitivity and specificity were calculated using two-sided 95% CI, computed by bootstrap method. Analyses were performed using the R package (R Core Team, 2020), version 4.0.2. It was estimated that a sample size of 1,535 plates would be needed to observe a two-sided 95% CI, computed using the score method, of width 0.04 plates for a target proportion of 0.8.

Cross tabulation and chi-square testing were performed using MedCalc Software (v.22.021) to examine potential differences in diagnostic performance outcomes across independent variables (e.g., across study sites) (10). *P*-values (significance threshold : *P* < 0.05) were utilized to determine whether the null hypothesis (no difference between independent variables) was accepted or rejected.

## RESULTS

**Fig 2:**
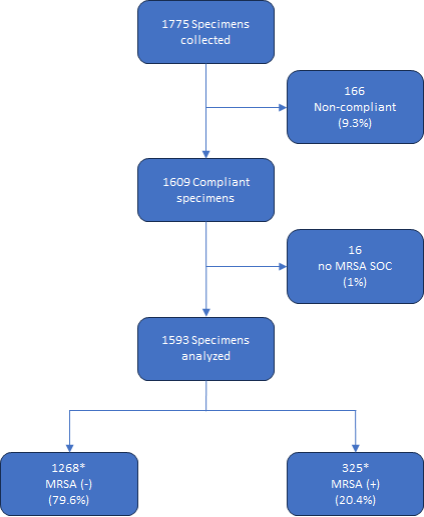
Reconciliation of plates assessed by reference method (laboratory technologist). *Of the 1,268 MRSA (−) specimens, 1,023 had no growth and 245 had no mauve growth; of the 325 MRSA (+) specimens, 319 were true positive and 6 were false negative. Abbreviations: SOC, standard of care; MRSA, methicillin-resistant *Staphylococcus aureus*.

Demographic data indicate that 3% of specimens were collected from patients less than 20 years of age, 27% were from patients between 20 and 59 years of age, 69% were from participants aged 60 and over, and there was no demographic information available for 1% of specimens. A total of 1,775 specimens were enrolled from the three participating sites, of which 166 were excluded for reasons such as time span of inoculation not covered by QC plates, missing incubation images, the algorithm-detected issues (e.g., bead in the plate or failed image quality check), and plates for which truth was not collected (e.g., plates discarded by a technologist, plate with no agreement), leaving 1,609 compliant specimens for evaluation. Of those 1,609 specimens, 16 cultures (1%) had no MRSA standard of care (SOC) results available and were therefore removed from further evaluation. By SOC, which is the medical or microbiology laboratory technologist reading, mauve-pigmented growth was detected in 325 (20.4%) of the 1,593 cultures, and no mauve-pigmented growth was detected in 1,268 (79.6%) of the plates assessed, including 1,023 plates with no-growth and 245 plates with non-mauve colony growth (Fig. 2). Of those, 1,545 (96.98%) were concordant between the MRSA App and SOC for the identification of MRSA colony growth [sensitivity 98.15% (95% CI, 96.03, 99.32) and specificity 96.69% (95% CI, 95.55, 97.60)]. At the level of individual study sites, Site 2 had a significantly lower sensitivity compared to Sites 1 and 3 while Site 3 had a significantly lower specificity compared to the other two sites (Table 1). Finally, the positive predictive value of the MRSA App for identifying MRSA-positive colonies was 88.11% (95% CI, 84.62, 90.89) and the negative predictive value was 99.52% (95% CI, 98.96, 98.78).

**TABLE 1:** Performance of MRSA application (index) compared to laboratory technologist score (reference) for detection of mauve (MRSA) colonies

Sites	N	TP	FN	FP	TN	Sensitivity [95% CI]	Chi-square for sensitivity	Specificity [95% CI]	Chi-square for specificity
Site 1	634	228	1	4	401	99.56% [97.59, 99.99]	8.47**^*a*, *b*^**	99.01% [97.49, 99.73]	10.03^*c*^**^, *d*^**
Site 2	634	42	4	13	575	91.30% [79.21, 97.58]	13.83**^*e*, *f*^**	97.79% [96.25, 98.82]	4.15^*g*^**^, *h*^**
Site 3	325	49	1	25	250	98.00% [89.35, 99.95]	0.01^*i*^**^, *j*^**	90.91% [86.87, 94.03]	36.59^*k*^**^, *l*^**
Total	1,593	319	6	42	1,226	98.15% [96.03, 99.32]	n/a	96.69% [95.55, 97.60]	n/a

^*a*^ Chi-square comparison of potential differences in FN and TP results from Site 1 versus Sites 2 and 3.

^*b*^ Indicates a *P*-value = 0.0036.

^*c*^ Chi-square comparison of potential differences in FP and TN results from Site 1 versus Sites 2 and 3.

^*d*^ Indicates a *P*-value = 0.0015.

^*e*^ Chi-square comparison of potential differences in FN and TP results from Site 2 versus Sites 1 and 3.

^*f*^ Indicates a *P*-value = 0.0002.

^*g*^ Chi-square comparison of potential differences in FP and TN results from Site 2 versus Sites 1 and 3.

^*h*^ Indicates a *P*-value = 0.0416.

^*i*^ Chi-square comparison of potential differences in FN and TP results from Site 3 versus Sites 1 and 2.

^*j*^ Indicates a *P*-value = 0.9301**^NS.^**

^*k*^ Chi-square comparison of potential differences in FP and TN results from Site 3 versus Sites 1 and 2.

^*l*^ Indicates a *P*-value < 0.0001.

Discrepant results were observed in 48 (3.01%) of the 1,593 cultures tested. The number of discrepant results was not equal across all testing sites. Hence, out of 634 specimens, two sites had 5 (0.8%) and 17 (2.7%) discrepancies, respectively, while the third site had 26 (8%) out of 325 specimens analyzed. Of these discrepant cultures, 42 were false positives in which the MRSA App detected mauve colonies (MRSA) while the microbiology technologists did not. Technologists found that 13 of these cultures had no growth, and 29 had growth of non-mauve colonies. Upon review of the images by an independent contributor as part of the discrepant analysis, 11 of the 42 cultures had very low amounts of mauve colonies (1–3 colonies) indicating that MRSA was present. Three agar plates contained areas of diffuse mauve coloring among heavy white colony growth. For the other 28 discrepant cultures, there was no obvious visible explanation as to why mauve growth was detected by the MRSA App. A total of six false-negative results were found, that is, plates in which MRSA was detected by the laboratory technologists and not the MRSA App (Table 1). Upon discrepant analysis, low amounts of mauve colonies (1–3 colonies) were identified in three of the cultures, indicating that MRSA was present (Fig. S1A through C). For one of these three plates, there was some non-mauve growth that may have confounded the MRSA App; the other two plates had only one apparent mauve colony with no non-mauve growth. There was no obvious visible growth (either mauve or non-mauve) that would explain the discrepancy for the other three cultures (Fig. S1D through F).

## DISCUSSION

This study evaluated the ability of the MRSA App to automate the detection of MRSA using digital images of MRSA chromogenic agar. Our reference standard was the laboratory technologist’s reading and interpretation of the same digital image. The MRSA App performed comparably with technologist reading of digital agar plate images, shown by 98.15% sensitivity and 96.69% specificity. This is in line with a study by Faron et al. which used PhenoMatrix automated imaging software in conjunction with WASPLab (Copan Inc., Murrieta, CA, USA) to detect MRSA from chromogenic agars from several manufacturers (11). In that study, BD CHROMagar MRSA had a sensitivity of 100% and a specificity of 90.7% which was similar to the other MRSA chromogenic agars evaluated in conjunction with PhenoMatrix. Automated image analysis programs for the detection of pigmented colonies on chromogenic agar have been evaluated for many organisms. As expected, overall, these studies have demonstrated high sensitivity with varying degrees of specificity (12–15).

Automated interpretation programs represent an opportunity to improve laboratory efficiency by reducing laboratory technologist labor associated with the review and interpretation of cultures. Staffing shortages are a major concern for clinical laboratories due to a low number of medical laboratory technologists entering the profession and an aging workforce. These challenges have become more severe during the SARS-CoV-2 pandemic and persist to this day. Implementation of laboratory automation equipment such as the Kiestra TLA and Copan WASPLab has increased the efficiency of clinical microbiology laboratories by eliminating repetitive work and allowing technologists to focus their skills on high-value work such as culture reading and interpretation (16–18). AI can be used to further reduce technologists’ hands-on time and increase laboratory efficiency. Using the MRSA App to sort MRSA screening cultures into growth/no growth categories, technologists can rapidly review and confirm pigmented colonies on chromogenic agar. MRSA screening cultures without growth can be reviewed in batches of up to 20 culture plates before generating final results. This process significantly reduces data entry in the laboratory information system, minimizes repetitive work, and may provide faster results to clinicians. In European laboratories, mauve colonies are reviewed by the technologist prior to reporting while no growth cultures are automatically released, and colonies with non-mauve growth are batch reported. This option removes all laboratory technologists’ intervention for cultures in which MRSA is not detected, thus helping to alleviate potential reporting delays, also enabling staff to focus on more complex tasks.

A careful review of digital images for the 48 discrepant cultures was performed to identify the reasons for the discrepant results. In all, 42 false-positive cultures were identified as MRSA growth by the MRSA App, but for which no mauve colonies were identified by the laboratory technologists. Sparse mauve colonies (1–3) were detected in 11 cultures, indicating the presence of MRSA. We observed that mauve colonies were often mixed with non-mauve colony growth, partially obscuring their presence and making them difficult to see by laboratory technologists. In three cultures, a diffused mauve pigment in the agar at the inoculation site possibly led to false-positive MRSA App results. In the other 28 cultures flagged by the App, no apparent visual rationale for the MRSA detection could be identified. However, algorithms are generally tuned to prioritize sensitivity over specificity, thus predisposing them toward “suspicion” when confidence levels hover around 0.5 (indicating an ambiguous level of certainty). This may explain as to the suboptimal decision of the App for those 28 results. Also, the priority toward higher sensitivity for the MRSA App impacts the positive predictive value of the MRSA App. Based on these results, one in ten positive results by the MRSA App would have the potential to initiate a patient management workflow that includes isolation, receipt of topical or systemic antibiotics, or other clinical attention that is not warranted due to a negative status. In this study, technologists were blinded to MRSA App results. However, under real-world circumstances in which technologists would not be blinded to the MRSA App results, all suspected MRSA-positive plates would be reviewed by the technologist (based on information from the MRSA App) to confirm positive colony status, thus reducing the occurrence of false-positive results and avoiding unnecessary clinical interventions. Therefore, the positive predictive value in a real-world clinical setting would likely be higher than the value determined in this study. Among the six false-negative cultures in which technologists identified MRSA but the App did not, three were confirmed with MRSA growth upon further evaluation. However, the basis for MRSA identification by technologists in the remaining three cultures, without observed mauve colonies, remains difficult to explain.

One limitation of the study was that the discrepant specimens were not available for repeat testing to ensure that the discrepancies were not merely imaging artifacts, or for confirmation of MRSA positivity by a different testing method. Another limitation pertains to the study design which allowed the technologists at each of the three sites to select one of two streaking patterns. Therefore, it is possible that the higher number of FP results observed at Site 3, compared to the other sites, was due to the streaking pattern selected. However, because specimens were de-identified, further analysis to determine a reason for this difference could not be performed.

### Conclusion

This multi-site study demonstrates that the MRSA App in conjunction with the BD Kiestra had 98.15% sensitivity and 96.69% specificity for detection of MRSA from anterior nares specimens. Through batch reading or auto-release of no-growth cultures, the BD Kiestra MRSA App can significantly shorten the laboratory technologist time associated with reading and interpretation of MRSA surveillance cultures. Improved workflows may also result in faster reporting of results to clinicians which rely upon MRSA surveillance results for enhanced infection control precautions, patient decolonization, and antimicrobial stewardship efforts.

## Data Availability

The data sets will be made available upon reasonable request to the corresponding author.

## SUPPLEMENTAL MATERIAL

Figure S1 jcm.01445-23-s0001.tif Images of agar plates with false-negative detection of MRSA by MRSA App.

ASM does not own the copyrights to Supplemental Material that may be linked to, or accessed through, an article. The authors have granted ASM a non-exclusive, world-wide license to publish the Supplemental Material files. Please contact the corresponding author directly for reuse.
