# Supplementary figures and images for: Automated detection of methicillin-resistant Staphylococcus aureus with the MRSA CHROM imaging application on BD Kiestra Total Lab Automation System
Source: J Clin Microbiol. 2024 Apr 1;62(5):e01445-23. doi: 10.1128/jcm.01445-23 (PMC11077980; doi:10.1128/jcm.01445-23)

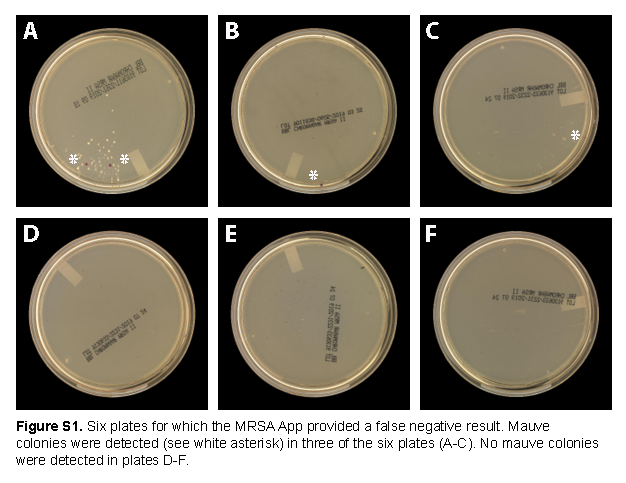

Supplement: Figure S1 — Images of agar plates with false-negative detection of MRSA by MRSA App. [file jcm.01445-23-s0001.tif]
